# Differential regulation of anti-inflammatory genes by p38 MAP kinase and MAP kinase kinase 6

**DOI:** 10.1186/1476-9255-11-14

**Published:** 2014-05-16

**Authors:** Deepa Hammaker, David L Boyle, Katharyn Topolewski, Gary S Firestein

**Affiliations:** 1 University of California San Diego School of Medicine, La Jolla, CA, USA

**Keywords:** p38 inhibitor, Rheumatoid arthritis, IL-10, MKK6, Tristetraprolin, Anti-inflammatory response

## Abstract

**Background:**

Conventional p38α inhibitors have limited efficacy in rheumatoid arthritis, possibly because p38 blockade suppresses the counter-regulatory mechanisms that limit inflammation. In contrast, targeting the upstream MAP kinase kinases, MKK3 and MKK6, partially maintains p38-mediated anti-inflammatory responses in bone marrow-derived macrophages (BMDM). In this study, we explored the mechanisms that preserve anti-inflammatory gene expression by evaluating differential regulation of IL-10 and p38-dependent anti-inflammatory genes in MKK3−/−, MKK6−/−, and p38 inhibitor-treated wildtype cells.

**Methods:**

BMDM from wild type (WT), MKK3−/−, and MKK6−/− mice were pre-treated with p38 inhibitor SB203580 (SB), JNK inhibitor SP600125 (SP), and/or ERK inhibitor PD98059 (PD) and stimulated with LPS. Supernatant protein levels were measured by multiplex bead immunoassay. mRNA expression was determined by qPCR and protein expression by Western blot analysis. De novo IL-10 mRNA synthesis was quantified in cells treated with ethynyl-uridine and LPS followed by reverse transcription and qPCR. mRNA half-life was measured in LPS-treated cells that were then incubated with actinomycin D ± SB203580.

**Results:**

Pre-treatment of WT BMDM with p38 inhibitor significantly reduced IL-10 production in the three groups, while ERK and JNK inhibitors had minimal effects. IL-10 production was significantly decreased in MKK3−/− BMDM compared with either WT or MKK6−/− cells. IL-10 mRNA expression was modestly reduced in MKK3−/− BMDM but was preserved in MKK6−/− cells compared with WT. De novo IL-10 mRNA synthesis was inhibited in MKK3−/− and p38 inhibitor pre-treated cells, but not MKK6−/− cells compared with WT. IL-10 mRNA half-life was markedly reduced in p38 inhibitor-treated WT cells while MKK-deficiency had minimal effect. DUSP1 mRNA levels were preserved in MKK-deficient cells but not in p38 inhibitor-treated WT cells. Tristetraprolin mRNA and protein levels were reduced in p38 inhibitor-treated WT cells compared with MKK6−/− cells.

**Conclusion:**

Unlike p38-inhibition, the absence of MKK6 mostly preserves IL-10 and TTP protein expression in BMDM. MKK6-deficiency also spares DUSP1 and IL-1RA, which are key negative regulators of the inflammatory response. Together, these data suggest that MKK6 is a potential therapeutic target in RA.

## Findings

### Background

Several highly specific p38α inhibitors that competitively bind the ATP binding pocket have been developed and evaluated in RA [1]. Despite promising pre-clinical data, these compounds show little therapeutic efficacy [2,3]. Several studies suggested that the unexpected lack of benefit in RA is due to the role of p38 in limiting inflammation, which might be blocked by the p38 inhibitor [4]. For instance, p38α down-regulates its own activity by inducing expression of dual specificity phosphatase 1 (DUSP1), which de-phosphorylates and inactivates p38 and JNK [5,6]. p38α also phosphorylates TAB1, leading to the inhibition of TAK1, a MAP3K that activates the p38 and JNK pathways [7]. p38α decreases MKK6 mRNA stability under basal conditions, providing negative feedback to its own signaling cascade [8]. Perhaps most relevant to the resolution of inflammation, p38α is required for the synthesis of IL-10, a potent anti-inflammatory cytokine that inhibits IL-6 and TNF expression [9] as well as Tristetraprolin (TTP), an RNA-binding protein that promotes the degradation of inflammatory cytokine mRNA [10,11]. Blocking these anti-inflammatory roles of p38α during and after inflammation might explain the lack of long-term efficacy of p38α inhibitors.

Our previous studies demonstrated that targeting either MKK3 or MKK6, which are the primary upstream activators of p38, might be superior to p38α blockade by preserving these anti-inflammatory responses. We recently showed that p38α^lysM^ mice, which lack p38α expression in macrophages, have increased arthritis severity in passive serum transfer and antigen-induced arthritis models [12]. In contrast, MKK3- or MKK6-deficiency reduces arthritis severity and joint destruction [13,14]. In addition, MKK3, but not MKK6, is required for optimal p38 activation in synovitis, whereas MKK6-deficiency is associated with lower IL-6, IL-17 and anti-collagen antibody production [14,15]. In bone marrow-derived macrophages (BMDM), p38 inhibition blocks IL-10 and DUSP1 expression while MKK-deficiency partially spared these anti-inflammatory responses [12]. It is not clear why the absence of p38α compared with MKK3 or MKK6 yields such divergent anti-inflammatory effects. In this study, we explored potential mechanisms by which p38 inhibition and MKK3- or MKK6-deficiency differentially regulates IL-10 production in BMDM. These data suggest that MKK6 is an attractive therapeutic target in the p38 pathway that preserves multiple p38-dependent anti-inflammatory pathways.

## Methods

### Bone marrow-derived macrophage culture (BMDM)

Bone marrow was isolated from the femur and tibia of DBA.1 WT, MKK3−/− and MKK6−/− mice and cultured in DMEM supplemented with 10% FCS and 20% L929-conditioned medium. After 7 days, the adherent BMDM were harvested, counted, and plated for use in experiments described below.

### Gene and protein expression

For gene expression assays, BMDM were treated with p38 inhibitor SB203580 (SB, 3 μM), JNK inhibitor SP600125 (SP, 20 μM) and/or ERK inhibitor PD98059 (PD, 100 μM) (Calbiochem) for 1 h prior to LPS stimulation (100 ng/ml, Invivogen). After 4 hours, mRNA was isolated and processed for quantitative PCR. Expression of DUSP1 and TTP was evaluated 1 h after LPS stimulation and IL-1RA after 4 h. For cytokine analysis, cells were stimulated with LPS for 24 h and the cell supernatants were assayed using multiplex immunoassay (Bio-Rad).

### Western blot analysis

BMDM were serum-starved overnight and stimulated with LPS (100 ng/ml) for various time points. The cells were lysed and 100 μg of lysate was subjected to SDS-PAGE. The proteins were transferred to a PVDF membrane and Western blot analysis was performed using anti-phospho p38 (Pp38) antibody (cat #9216, Cell Signaling Technology), total p38 antibody (cat #8690, Cell Signaling Technology), anti-β actin (cat #sc-1616, Santa Cruz Biotechnology) and anti-TTP antibody (a kind gift of Dr. Perry Blackshear, National Institute of Environmental Health Sciences, North Carolina, US).

### mRNA decay

After 4 h of LPS stimulation, WT (n = 6), MKK3−/− (n = 3), and MKK6−/− (n = 3) BMDM were treated with actinomycin D (10 μg/ml) for various times and IL-10 mRNA levels were analyzed by qPCR (Applied Biosystems) and normalized to β-actin. To determine the effect of p38 inhibitor on mRNA decay, WT cells were treated with SB (3 μM) after LPS stimulation (WT SB post) and mRNA expression was assayed as described above.

### Nascent RNA transcription rate

De novo mRNA synthesis was measured in WT, MKK3−/−, and MKK6−/− BMDM treated with ethynyl-uridine with or without LPS for 1 h. The RNA was quantified and biotinylated using Click-iT Nascent RNA capture kit (Invitrogen). Nascent RNA was isolated using streptavidin-magnetic beads. Reverse transcription was performed using SuperScript VILO cDNA synthesis kit (Invitrogen) and IL-10 expression was measured by qPCR and presented as fold of LPS-treated WT cells.

### Statistical analysis

Comparisons between WT, MKK3−/−, and MKK6−/− cells were analyzed by two-way or one-way ANOVA and Tukey or Dunnett multiple comparison tests, unless otherwise stated. Data were analyzed using GraphPad Prism 6.0 and the comparisons were considered statistically significant if *p* < 0.05.

## Results

### Regulation of IL-10 protein and gene expression in MKK-deficient or p38 inhibitor- treated BMDM

**Figure 1:**
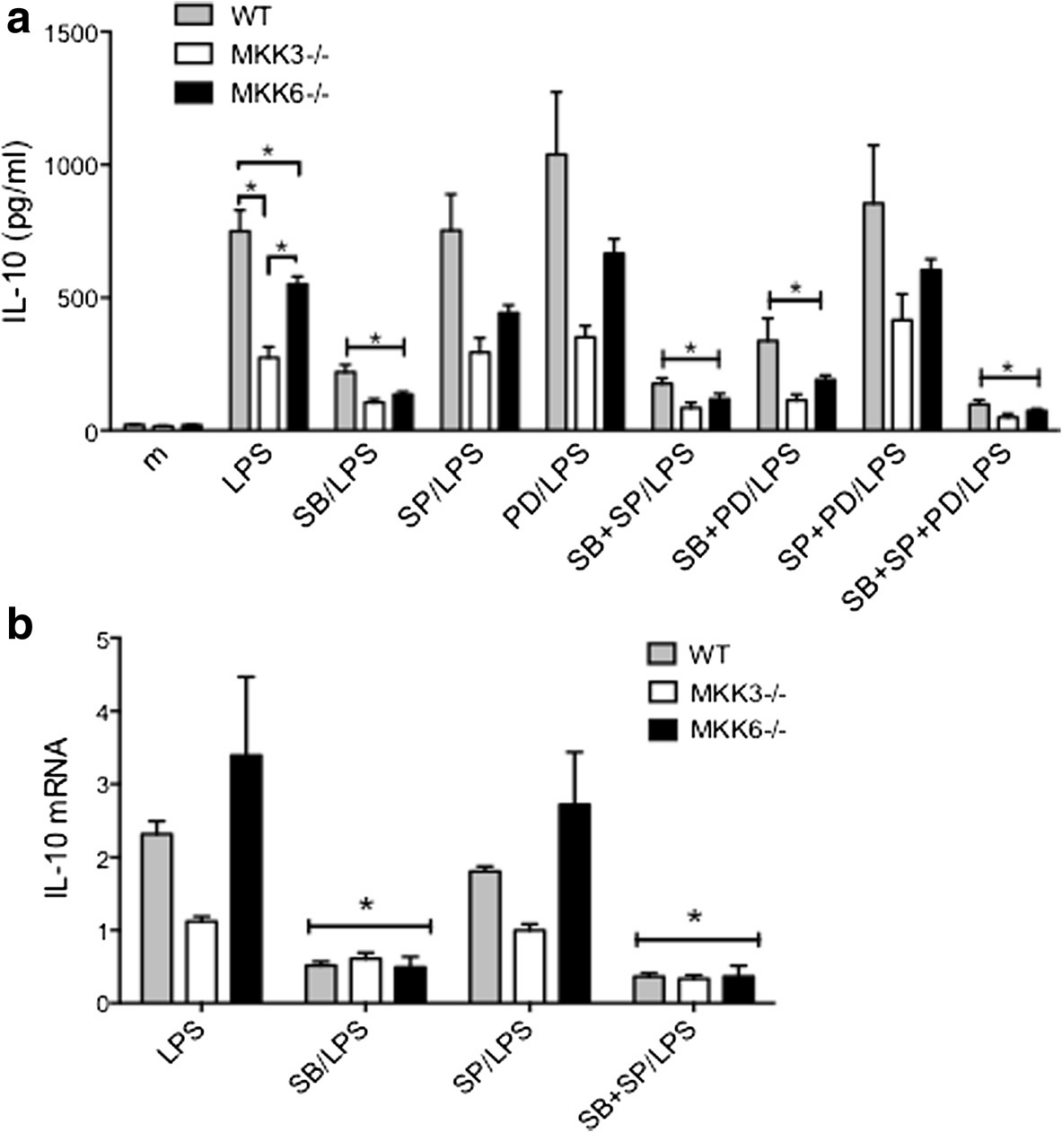
**Regulation of IL-10 protein and gene expression in MKK-deficient or p38 inhibitor-treated BMDM. (a)** IL-10 protein levels were significantly reduced in MKK3−/− compared with MKK6−/− or WT cells. MKK6-deficiency partially preserved IL-10 expression compared with WT cells. Treatment with the p38 inhibitor significantly decreased IL-10 production in all three groups while JNK or ERK inhibition showed no major effects (*p < 0.05). **(b)** IL-10 mRNA expression was modestly reduced in MKK3−/− cells compared with WT or MKK6−/− cells. As with protein expression, p38 inhibitor reduced IL-10 mRNA expression in WT, MKK3−/− and MKK6−/− cells but JNK inhibitor showed minimal effect.

We first evaluated IL-10 production in LPS-stimulated WT, MKK3−/− and MKK6−/− BMDM and compared it to cells pre-treated with the p38 inhibitor (SB) (Figure 1a). IL-10 protein expression was induced by LPS but was significantly lower in MKK3−/− (p < 0.0001, n = 3/group) compared with either WT or MKK6−/− BMDM. MKK6−/− cells had only modest reduction in IL-10 levels compared with WT cells. As expected, the p38 inhibitor significantly reduced IL-10 in all three groups compared with their respective LPS controls while JNK and ERK inhibitors had no significant effect on LPS-induced IL-10 production by WT, MKK3−/− and MKK6−/− cells. Combination of SP and/or PD with SB did not rescue IL-10 production in SB-treated cells. IL-10 mRNA expression was modestly reduced in MKK3−/− compared with WT or MKK6−/− cells (Figure 1b). However, p38 inhibition significantly decreased IL-10 mRNA expression in WT, MKK3−/− and MKK6−/− BMDM while JNK blockade did not have a major effect. These results show that p38, but not JNK or ERK, regulates IL-10 production in BMDM. More importantly, deficiency of MKK6 and, to a lesser extent MKK3, partially maintains IL-10 expression compared with direct p38 inhibition.

### Transcriptional regulation of IL-10 in MKK-deficient BMDM

**Figure 2:**
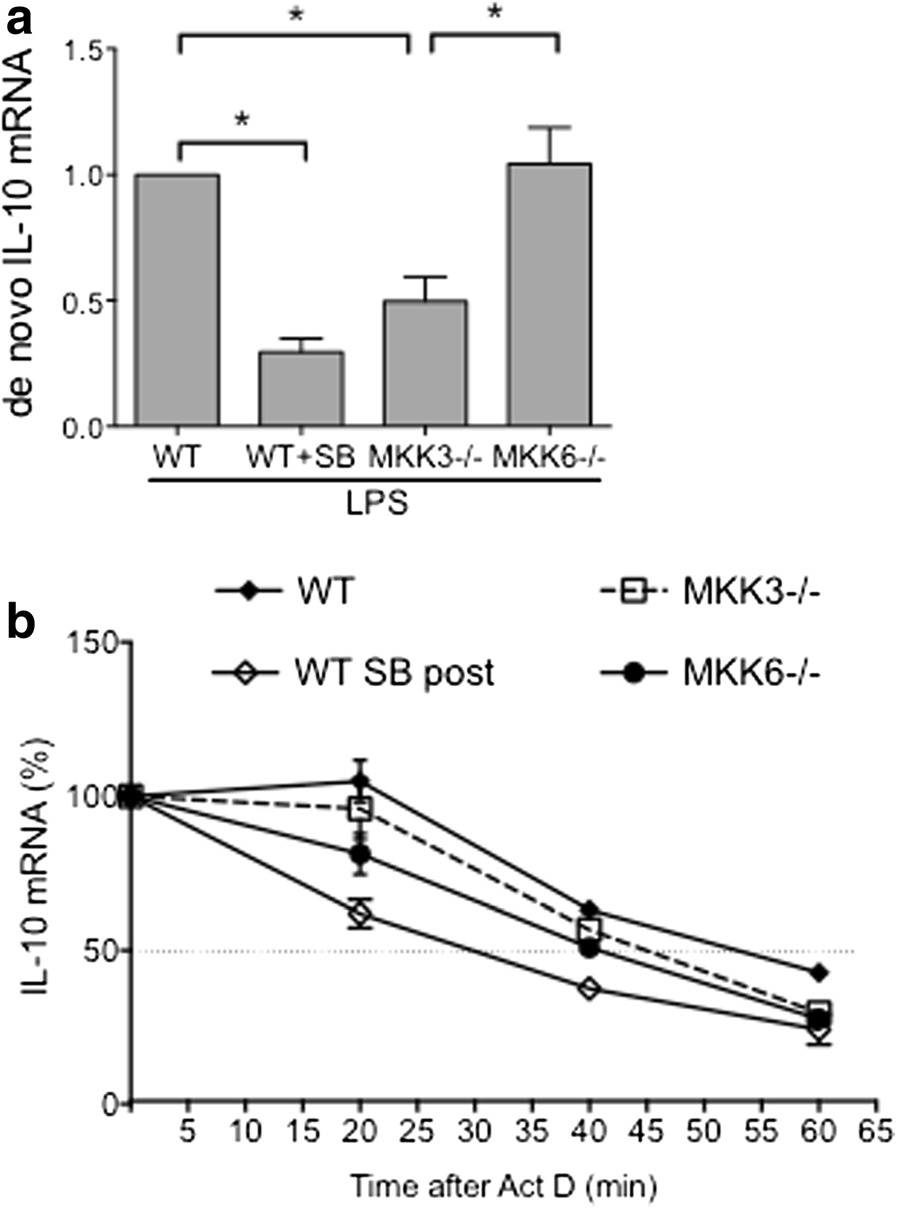
**Transcriptional and post-transcriptional regulation of IL-10 expression. (a)** De novo IL-10 transcription rate was significantly inhibited in p38 inhibitor-treated WT and MKK3−/− cells compared with WT control. MKK6−/− cells expressed IL-10 at a rate comparable to WT control (*p < 0.05). **(b)** IL-10 mRNA decay in MKK-deficient cells was comparable to WT control. p38 inhibitor-treated WT cells showed a significantly higher rate of IL-10 degradation compared with WT control.

Since IL-10 expression is regulated transcriptionally and post-transcriptionally, the effect of MKK-deficiency or p38 inhibition on IL-10 transcription rate was evaluated. Labeled nascent mRNA from p38 inhibitor-treated WT cells and MKK3−/− or MKK6−/− cells was isolated, and assayed by qPCR (Figure 2a). The p38 inhibitor as well as MKK3-deficiency significantly decreased de novo LPS-induced IL-10 transcription rate compared with WT LPS control. However, IL-10 transcription was comparable in MKK6−/− cells and WT cells, which is consistent with our gene expression and protein data.

### Post-transcriptional regulation of IL-10 in MKK-deficient BMDM

IL-10 mRNA transcript stability in MKK-deficient cells was determined by comparing IL-10 decay rates in WT, MKK3−/−, and MKK6−/− cells (Figure 2b). IL-10 mRNA decay was rapid, with half-life of 40 minutes for MKK6−/−, 46 minutes for MKK3−/− and 54 minutes for WT BMDM. In WT cells treated with p38 inhibitor (WT SB post), IL-10 mRNA half-life of 30 minutes was significantly lower than in WT cells. These data suggest that unlike p38 inhibition, IL-10 expression in MKK3- or MKK6-deficient BMDM is primarily regulated at the transcriptional level but not by altering mRNA degradation.

### Effect of p38 inhibitor or MKK-deficiency on the expression of other anti-inflammatory molecules

**Figure 3:**
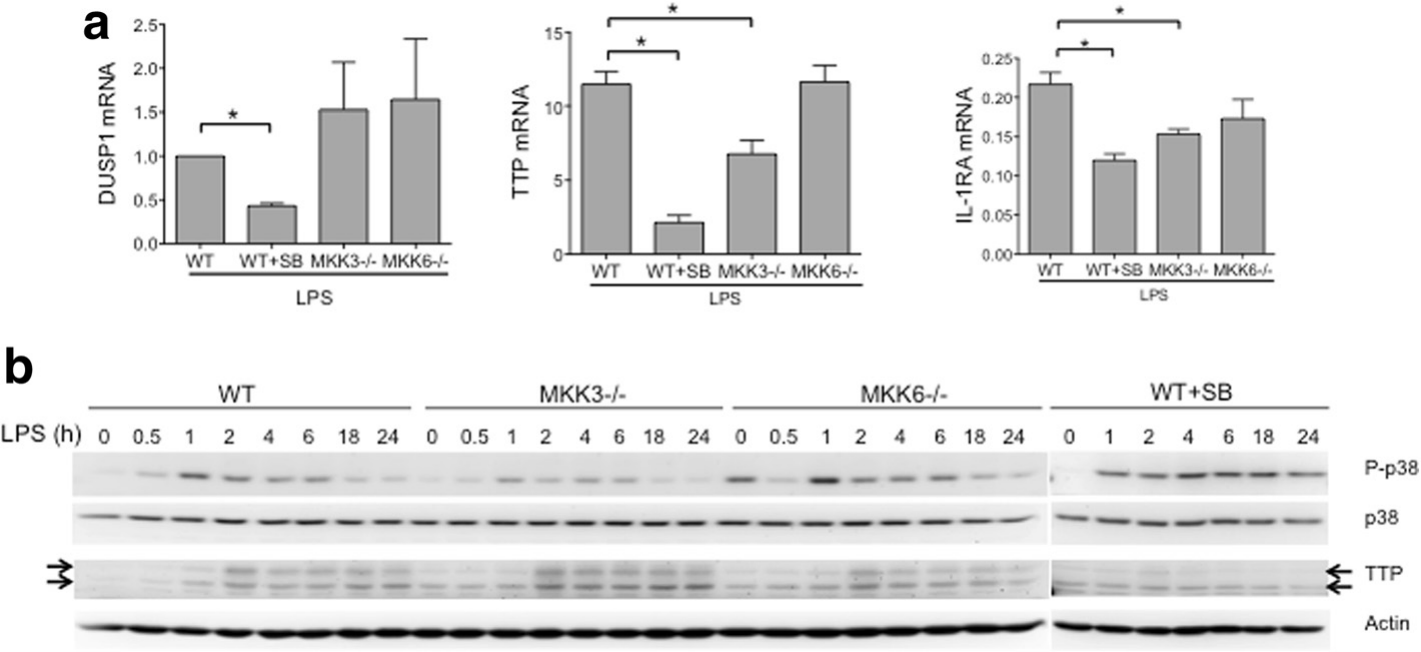
**Regulation of other p38-dependent anti-inflammatory genes. (a)** DUSP1 mRNA expression was significantly reduced in p38 inhibitor-treated WT cells compared with WT control but not in MKK-deficient cells (*p < 0.05). TTP and IL-1RA mRNA levels were significantly reduced in the presence of the p38 inhibitor and MKK3−/− cells but not MKK6−/− cells compared with WT. **(b)** Western blot analysis showed reduced p38 phosphorylation in MKK3−/− BMDM but not MKK6−/− BMDM. TTP protein expression was similar in WT, MKK3−/− and MKK6−/−, although it was modestly elevated in MKK3−/− cells.

We then determined if the effects of MKK-deficiency on IL-10 could be generalized to the expression of other anti-inflammatory genes associated with p38 pathway. As expected, DUSP1 expression in p38 inhibitor-treated WT group was significantly lower than control (p = 0.005, n = 3, Figure 3a). In contrast, DUSP1 expression in MKK3−/− and MKK6−/− cells was comparable to WT. Expression of tristetraprolin (TTP) and IL-1 receptor antagonist (IL-1RA) mRNA was significantly reduced in p38 inhibitor-treated and MKK3−/− cells compared with WT, while MKK6−/− expressed WT levels of both genes. Together, these data show that p38 inhibition, but not MKK6 deficiency, significantly blocks DUSP1, IL-1RA and TTP expression. Since TTP is an important negative regulator of proinflammatory cytokines, we next evaluated the effect of MKK-deficiency and p38 inhibition by Western blot analysis (Figure 3b). P-p38 levels were lower in MKK3−/− cells compared with WT or MKK6−/− cells. Consistent with our previous studies, MKK3 is the primary kinase for phosphorylating p38 in BMDM [12]. Although MKK6 is not required for p38 phosphorylation, it is necessary for the efficient induction of cytokines such as IL-6 in BMDM, indicating that MKK6-deficiency has effects downstream of p38 phosphorylation (Additional file 1: Figure S1) [16]. TTP expression peaked after 2 h of exposure to LPS in the WT and MKK−/− groups, although surprisingly, MKK3−/− cells showed modestly higher levels of TTP. Others have shown that TTP degradation is mediated by phosphorylation of MAPKAPK2 by p38 [11]. It is likely that reduced P-p38 levels in MKK3−/− cells reduces MAPKAPK2 phosphorylation leading to elevated TTP levels. Expression of TTP was markedly decreased in p38 inhibitor treated WT cells, as shown previously [10]. Together, these data show that p38 inhibitor and MKK6-deficiency can differentially regulate TTP mRNA and protein in BMDM.

## Conclusions

The p38 pathway regulates the resolution of inflammatory responses by increasing expression of anti-inflammatory cytokines to promote wound healing and homeostasis. Blocking p38 inhibits the expression of IL-10 and p38-dependent anti-inflammatory genes such as DUSP1, TTP, and IL-1RA. In contrast, MKK6-deficiency allows partial or full induction of IL-10 and TTP, which negatively regulate the inflammatory cascade. These data suggest that blocking MKK6 might be a potential therapeutic target for inflammatory diseases such as RA that avoids some of the limitations of a conventional p38 inhibitor.

## Abbreviations

MAP: Mitogen activated protein kinase; MKK: MAP kinase kinase; JNK: cJun N-terminal kinase; BMDM: Bone marrow derived macrophages; IL-10: Interleukin 10; WT: Wild type; SB: SB203580 (p38α/β inhibitor); SP: SP600125 (JNK inhibitor); PD: PD98059 (ERK inhibitor); LPS: Lipopolysaccharide; qPCR: Quantitative PCR; DUSP1: Dual specificity phosphatase 1; IL-1RA: IL-1 receptor antagonist; TTP: Tristetraprolin; RA: Rheumatoid arthritis; TAB1: TAK1 binding protein 1; TAK1: TGFβ-activated kinase 1; MAP3K: MKK kinase; TNF: Tumor necrosis factor; MAPKAPK2: MAP kinase activated protein kinase 2.

## Competing interests

The authors declare that they have no competing interests.

## Authors’ contributions

DH designed and performed the experiments, analyzed data and prepared the manuscript. DLB contributed to the data analysis and manuscript preparation. KT performed experiments. GSF conceived and designed the experiments, analyzed the data and prepared the manuscript. All authors read and approved the final manuscript.

## Supplementary Material

Additional file 1: Figure S1 Effect of MKK3- and MKK6-deficiency on IL-6 promoter activity. WT, MKK3−/− and MKK6−/− BMDM were transfected with 2 μg of IL-6 promoter construct (pGL4.10-IL-6/luc, a kind gift of Dr. Peter Sporn, Northwestern University, Chicago, IL) and 0.2 μg of Renilla construct and stimulated with 100 ng/ml LPS for 24 h. The cells were lysed and the luciferase activities were measured using Dual luciferase reporter assay system (Promega). The ratio of firefly/renilla luciferase was determined for 3 different BMDM lines/group. The data are represented as average fold of WT LPS. Click here for file
