# Supplementary figures and images for: Differential regulation of anti-inflammatory genes by p38 MAP kinase and MAP kinase kinase 6
Source: J Inflamm (Lond). 2014 May 16;11:14. doi: 10.1186/1476-9255-11-14 (PMC4030013; doi:10.1186/1476-9255-11-14)

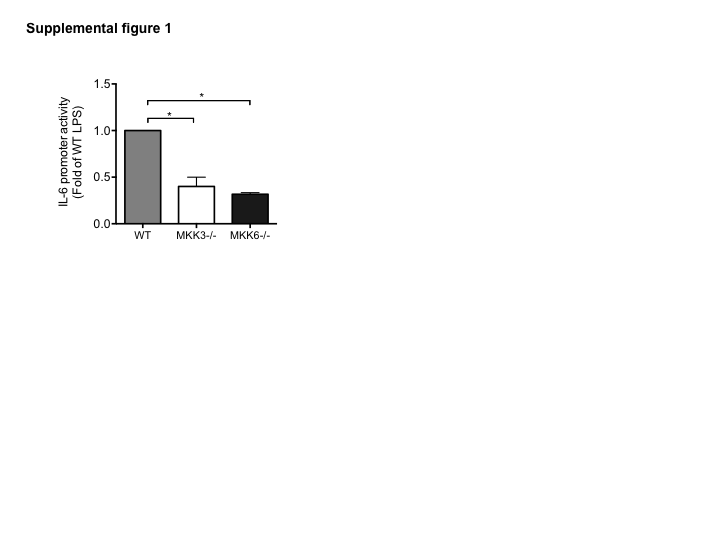

Supplement: Additional file 1: Figure S1 — Effect of MKK3- and MKK6-deficiency on IL-6 promoter activity. WT, MKK3−/− and MKK6−/− BMDM were transfected with 2 μg of IL-6 promoter construct (pGL4.10-IL-6/luc, a kind gift of Dr. Peter Sporn, Northwestern University, Chicago, IL) and 0.2 μg of Renilla construct and stimulated with 100 ng/ml LPS for 24 h. The cells were lysed and the luciferase activities were measured using Dual luciferase reporter assay system (Promega). The ratio of firefly/renilla luciferase was determined for 3 different BMDM lines/group. The data are represented as average fold of WT LPS. [file 1476-9255-11-14-S1.tiff]
